# The Impact of the Integrated Development of AI and Energy Industry on Regional Energy Industry: A Case of China

**DOI:** 10.3390/ijerph18178946

**Published:** 2021-08-25

**Authors:** Feng Dong, Shengnan Zhang, Jiao Zhu, Jiaojiao Sun

**Affiliations:** School of Economics and Management, China University of Mining and Technology, Xuzhou 221116, China; ts17070109a3@cumt.edu.cn (S.Z.); ts20070019a31@cumt.edu.cn (J.Z.); cumtsjj@126.com (J.S.)

**Keywords:** artificial intelligence, energy industry, synthetic control method, integrated development

## Abstract

With the advent of the Energy 4.0 era, the adoption of “Internet + artificial intelligence” systems will enable the transformation and upgrading of the traditional energy industry. This will alleviate the energy and environmental problems that China is currently facing. The integrated development of artificial intelligence and the energy industry has become inevitable in the development of future energy systems. This study applied a comprehensive evaluation index to the energy industry to calculate the comprehensive development index of the energy industry in 30 provinces of China from 2000 to 2017. Then, taking Guangdong and Jiangsu as examples, the synthetic control method was used to explore the direction and intensity of the integrated development of artificial intelligence and the energy industry on the comprehensive development level of the local energy industry. The results showed that when artificial intelligence (AI) and the energy industry achieved a stable coupled development without the need to move to the coordination stage, the coupling effect promoted the development of the regional energy industry, and the annual growth rate of the comprehensive development index was above 20%. This coupling effect passed the placebo test and ranking test and was significant at the 10% level, indicating the robustness and validity of the experimental results, which strongly confirmed the great potential of AI in re-empowering traditional industries from the data perspective. Based on the findings, corresponding policy recommendations were proposed on how to promote the development of inter-regional AI, how the government, enterprises, and universities could cooperate to promote the coordinated development of AI and energy, and how to guide the integration process of regional AI and energy industries according to local conditions, in order to maximize the technological dividend of AI and help the construction of smart energy in China.

## 1. Introduction

As a pillar industry of the national economy, the development and planning of energy generation will shape the future development and trends of the national economy, society, and livelihoods. With the rapid development of industrial technology and information technology, a new energy utilization system will deeply integrate new energy technology and information technology, enabling the development of the energy system to be clean and intelligent. The fourth industrial revolution, with the energy Internet as the core feature, will finally arrive, and the energy system will enter the Energy 4.0 era [1,2]. Energy 4.0 is not only the use of the Internet system to transmit energy, but also the use of Internet technology to coordinate the use and efficiency of various energy sources, forming a multi-level correlation system that includes information space, physical space, energy space, and social space, so that the differences between different energy sources can be balanced and the advantages of different energy sources can be maximized to form a cumulative effect [3]. The entropy increase tendency of energy leads to less and less energy use. Therefore, it is necessary to make full use of various energy sources with the help of advanced industrial technologies and to adopt a multi-energy complementary model in order to achieve a secure energy supply. In turn, Energy 4.0 reshapes the economic structure and provides clean, environmentally friendly, low-consumption green energy for the fourth industrial revolution [4,5,6].

Artificial intelligence (AI) being a method of simulating the biological human brain in order to complete tasks in a more effective manner could be used in various applications. By simulating the cognitive abilities of the human brain, AI could be used to respond to input in a manner similar to that of humans [7]. A White Paper on the application of artificial intelligence in China, jointly published by the Chinese artificial intelligence society and Roland Berger in 2017, provided an in-depth analysis of the concept of artificial intelligence [8]. Artificial intelligence is defined as the science of simulating a series of human intelligent behaviors, such as autonomous learning, decision making, and judgments. The “Internet + artificial intelligence” may become a key breakthrough for China’s energy system to achieve optimization, transformation, and upgrading and help the construction of smart energy, effectively solve the problem of resource scheduling and optimal allocation on the wide-area energy network, alleviating China’s energy and environmental problems. The integrated development of artificial intelligence and the energy industry has become an inevitable trend in the development of future energy systems. The development of artificial intelligence in China is extremely geographically uneven, with large differences across the country in the integration and development of artificial intelligence in the energy industry. An in-depth understanding and objective analysis of the development status and integration process of AI and energy industry at the regional level is a prerequisite for accelerating the effective integration of AI and energy industry. Theoretically, technological innovation can promote industrial development, but it is still unknown whether the integration of AI and energy industry at this stage can promote the transformation and upgrading of the local energy industry and inject fresh development momentum into the local energy industry. There is no quantitative scientific research on whether the integration of artificial intelligence, an emerging field with high risks and high opportunities, is necessary with the traditional energy industry. This paper will try to answer these questions from the perspective of data. Only by clarifying the correctness and necessity of the integration and development of AI and energy industry and putting forward targeted policy recommendations and action measures according to the actual development status of each region can we effectively promote the construction of smart energy in China.

Most of the existing literature on AI and smart energy are separate and independent within each field or focused on the application and development of AI in the traditional energy industry, with more emphasis on the technical aspects [9,10]. There is a gap in academic research on the relationship between AI and energy industry from a macro perspective, and lack of quantitative analysis to measure the relationship between them. This paper explores the degree of influence of the integration development of AI and energy industry on the comprehensive development level of regional energy industry in the regions where the coupling development of AI and energy industry has been achieved during the sample period. The main contributions are as follows: (1) some regions have achieved the coupling and coordinated development of AI and energy industry, but whether this integration development has a positive promoting effect or a negative inhibiting effect on the local energy industry is unknown. It is also unknown when this coupling effect starts to work and how much it affects the local energy industry, but this paper can quantify this coupling effect. (2) This paper uses the synthetic control method to compare the synthesized energy industry comprehensive development index with the actual value, which is beneficial to a more intuitive and profound understanding of the coupling effect and strength of the integration for AI and energy industry. (3) The placebo test and ranking test are applied to verify the robustness and validity of the results of the synthetic control method and demonstrate the huge potential of artificial intelligence in empowering traditional industries.

This study proceeds as follows. Section 2 is the literature review that presents the research on AI, smart energy in “Internet+” mode, and the theoretical foundation of the synthetic control method. Section 3 displays the model construction, and the details of our data and variables. Empirical results and analysis are presented in Section 4. Section 5 concludes and gives some relevant policy recommendations.

## 2. Literature Review

### 2.1. Research on AI

As an important branch of computer science, the core purpose of artificial intelligence is to use machines to simulate the thinking process of human beings, and then replace human beings to complete the corresponding work and realize the automation of intelligent behavior [11]. In practical applications, artificial intelligence refers to changes in the environment where a machine perceives text, sound, images, and definable symbols for input, and according to pre-set rules, the target task is automatically executed by the relevant algorithm [12]. With the establishment and sharing of large-scale data platforms, data mining technologies and deep learning algorithms have improved computing power, and sensor system technologies, such as processors, sensors, and chips have developed rapidly [13]. The theory and industrial application of artificial intelligence have developed rapidly in the past 30 years.

Artificial intelligence has fully entered the machine learning era and has made breakthroughs in applications such as intelligent robots, intelligent drones, automatic driving, intelligent interactions, natural language processing, and computer vision and images. It has realized technological upgrades in many traditional industries such as finance, culture and education, medical treatment, electric power, petrochemicals, and mining [14], which has triggered innovation in the digital transformation of traditional industries and the real economy, the optimization of industrial chain structure, information processing, and personnel arrangement efficiency. Artificial intelligence has passed from the theoretical research era to the commercial stage. Studies using industry panel data in 17 countries around the world over the past 15 years have shown that industrial robots can greatly improve labor productivity and economic growth [15]. In the financial field, artificial intelligence has enabled a number of financial services to subvert the traditional form of financial services to help the financial industry achieve high-precision financial product marketing and low-cost risk control. A rule-based expert system was established to help financial institutions make quick and accurate judgments on the authorization of enterprise credit loans [16]. Using a model based on the back propagation (BP) neural network algorithm, the credit rating of China’s Taiwan market and the US market were analyzed [17]. The accuracy of credit risk classification in Australia and the United Kingdom has been improved by applying a fuzzy approximate support vector machine model for the risk assessment of credit data sets [18]. Whiting et al. studied financial fraud detection based on machine learning and deep learning [19]. A new cost forecasting model based on artificial intelligence technology was built, which improved the accuracy of cost forecasting by 95% [20].

Artificial intelligence has also had a revolutionary impact on the medical and health field, promoting the reform and innovation of medical technology, as well as the optimization and upgrading of the medical service mode. Through machine learning, natural language processing, and other technologies, an intelligent diagnosis of medical conditions can be made, and personalized diagnosis and treatment schemes can be provided. Patients will receive an intelligent diagnosis and be given a personalized diagnosis and treatment plan, which will substantially shorten their treatment time, improve their satisfaction with the medical treatment, and reduce potential conflicts between doctors and patients [21,22]. At the same time, artificial intelligence also has great potential in disease prevention and drug research and development [23,24,25,26,27,28,29]. Educational artificial intelligence is an emerging field combining artificial intelligence with learning science [30]. It aims to observe and explain the learning process and the mechanisms by which external factors interfere with the learning process from a micro perspective through the application of artificial intelligence technology [31]. The application of artificial intelligence in the education industry is still within the auxiliary function of the learning process. The integration of artificial intelligence and the manufacturing industry is mainly manifested in three application scenarios: intelligent product development and design, intelligent quality inspection, and the predictive maintenance of production equipment. In the field of government services, AI-related technologies such as natural language processing, facial recognition, and machine learning can provide unmanned government services such as intelligent identity authentication, accurate information retrieval, and online intelligent customer service [32].

### 2.2. Research on Smart Energy in “Internet+” Mode

Although China only officially proposed the development goal of “Internet+” intelligent energy in 2015, the research of energy system intelligence has been long-standing. The development and improvement of artificial intelligence-related technologies and industries have continuously promoted the integration of artificial intelligence with energy systems, and its research and application in coal mines, petroleum, and electric power are becoming increasingly mature.

In the coal field, the traditional production and operation mode and safety hazards have seriously restricted the survival and development of the coal industry, and it has become imperative to realize the intelligence of the coal industry. Wang et al. studied the intelligent mining technology, safe and efficient coal general mining technology, and intelligent working face technology in the coal industry, and proposed the concept of “smart mine” in 2018, which pointed out the direction for the intelligent development of the coal industry in the future [33,34,35]. Yan et al. [36] proposed a hybrid artificial intelligence model combining a BP neural network (BPNN), genetic algorithm (GA), and adaptive boosting algorithm (AdaBoost), which could better evaluate the strength alteration of coal during CO_2_ geological sequestration. In addition, artificial intelligence algorithms such as support vector machines and neural networks are often used by experts and scholars in the coal field for the deep mining of coal data information and training models [37,38].

The application of artificial intelligence in the oil industry involves oil and gas exploration, transmission, mining, sales, and the operating processes that link all these stages. It provides intelligent services for related scenarios, such as the use and maintenance of oil extraction equipment, intelligent diagnosis, security and early warning of oil transmission equipment failure, prediction, and optimization of oil extraction schemes, and prediction of oil resources. Based on downhole and production data, an intelligent decision-making platform for evaluating the development value of oil and gas resources was constructed [39]. Using natural language processing technology, an automatic question-and-answer system was developed to provide relevant training to oil and gas developers 24 h a day [40]. Khan et al. used deep learning, a neural network, and other technologies to predict the oil recovery rate of artificial gas lift wells, with an accuracy of up to 99% [41]. Some of the advantages of using neural network models are that it does not require any a priori assumptions about the dependence of the functional form of the underlying process and can also be used to establish relationships between complex nonlinear data problems by providing numerical models and can reduce noise in the data [42].

The smart grid is the latest trend in the development and reform of the world’s power system, and its effectiveness relies on the in-depth and extensive application of artificial intelligence technology in the power system. A migration learning algorithm is introduced into the power dispatching system, and the automatic dynamic distribution of power generation control power is realized through the analysis of historical data [43]. To avoid power consumption and latency, Qaisar [44] proposed a new approach that combined signal-guided acquisition, adaptive rate segmentation, and time-domain feature extraction with machine learning tools to reduce the computational cost and latency of the classifier. The structure of power grids has become increasingly complex, with many uncertain factors having increasing effects on grid systems. Therefore, when developing a smart grid, it is of great significance to develop an effective risk assessment method for the transmission system to ensure its safety and stability. 

### 2.3. The Synthetic Control Method

The synthetic control method is developed based on a counterfactual estimation framework. Abadie and Gardeazab [45] first proposed the synthetic control method by applying it to identify the economic costs of terrorist activities in the Basque Country of Spain. Abadie [46] applied the synthetic control method to evaluate the effects of the California tobacco control law enacted in 1998. Abadie [47] again used the method to estimate the economic impact of German reunification in 1990 on Western Germany. Synthetic controls provide many practical benefits for estimating the impact of policy interventions and other related events [48].

Synthetic control methods have been used to identify the effects of a policy. The synthetic control method was used to study the impact of Arizona’s 2007 Legal Arizona Workers Act on illegal immigration to the state, and the results indicated that the Act had a significant impact on illegal immigration [49]. The methodology was also used to study the impact of economic liberalization on real GDP in countries around the world. Liberalization was found to have an impact in most regions [50]. The synthetic control method confirms the significant economic boosting effect of the establishment of the Shanghai Free Trade Zone [51]. Liu and Lv [52] conducted a comparative analysis of the agglomeration effects of the four early FTAs established in China, and the synthetic control method showed that the establishment of the FTAs all produced positive economic effects and showed obvious differentiation characteristics. Li and Li [53] explored the economic effects of economic zone planning on the western region of China through the synthetic control method.

Some regions have achieved coupled and coordinated development of AI and energy industries. However, it is still unknown whether and when this convergent development has a positive or negative effect on the local energy industry and the exact magnitude of this coupling effect. This paper addresses these questions using a synthetic control method popular in the field of evaluating policy effects.

## 3. Model Construction and Variable Selection

### 3.1. Model Construction

The synthetic control method is a newly emerged method of analysis based on natural experiments in comparative case analysis, proposed by Abadie and Gardeazabal [45]. In recent years, the method has been continuously improved [46,47,48]. The synthetic control method is mainly used to identify the implementation effect of a certain policy. The basic idea is that due to the heterogeneity of each economy, it is difficult to find an economy with essentially the same attributes and characteristics as the experimental group as a reference group in a realistic scenario, but several known reference groups can be linearly combined into a “composite group” that can better match the basic characteristics of the experimental group. This synthetic control object is the “counterfactual reference group” of the experimental group to be studied without policy interference simulated by the experiment. By comparing the difference between the actual experimental group and the synthetic experimental group, it is possible to know whether the policy under study is effective. This study used the synthetic control method, which is commonly used to evaluate the effects of policy. A comparative experimental study was conducted by considering a region where the coupling of artificial intelligence and energy had been achieved. The development of the local energy industry was considered under two scenarios by comparing a system in which artificial intelligence and the energy industry had been adopted to achieve the coordinated development of coupling with a system without coupling and coordinated development. The result of the comparison revealed the impact of the coupling of the two systems on the development of the energy industry in the study region.

It was assumed that the comprehensive development level of the energy industry in *N* + 1 provinces could be observed within statistical time [1, *T*]. The first province (experimental group) in the *T_0_* stage achieved the coupling of artificial intelligence and the energy industry, while the other *N* provinces did not realize the coupling of artificial intelligence and the energy industry, or local artificial intelligence did not develop during the statistical period. Then, *N* provinces could be used as a potential reference group for experimental province 1. 

$Y_{1it}$ represents the comprehensive development index of the energy industry in province *i* when regional artificial intelligence and the energy industry are coupled in period *t*. $Y_{0it}$ represents the observation result of the comprehensive development level of the regional energy industry in province *i* without realizing the coupling of artificial intelligence and the energy industry in period *t*. Therefore, the effect in region *i* was caused by the coordinated development of artificial intelligence and the energy industry during the period *t*: $\tau_{it}=Y_{1it}-Y_{0it}$, where i=1,···,N+1; t=1,···,T. According to the above formula, the comprehensive development level of the regional energy industry observed in *i* province during *t* period can be expressed as: $Y_{it}=D_{it}Y_{1it}+(1-D_{it})Y_{0it}=Y_{0it}+\tau_{it}D_{it}$. Among them, $D_{it}$ is a dummy variable, representing the integrated development state of artificial intelligence and the energy industry of province *i* in period *t*. If province *i* realized the coupling of artificial intelligence and the energy industry in period *t*, the value of $D_{it}$ was 1, and vice versa.

Only the first province realized a coupling between artificial intelligence and the energy industry after *T_0_*, while the other *N* provinces did not realize the coupling and coordinated development of regional artificial intelligence and the energy industry at any time in the statistical period. For $t>T_{0}$ period, the coupling effect of the coordinated development of the two systems in the first province on the comprehensive development level of the regional energy industry can be expressed as: $\tau_{1t}=Y_{11t}-Y_{01t}=Y_{1t}-Y_{01t}$. The aim of this study was to solve $\tau_{1t}$. Because the first region realized the coupling and coordinated development of the two systems, $Y_{11t}$ could be observed in the $t>T_{0}$ period. The comprehensive development level $Y_{01t}$ of the energy industry in the first province without the coupling of the two systems was not observed. The model (1) below was used to calculate the “counterfactual”result $Y_{01t}$ [46]. The comprehensive development level of the local energy industry in the first province was estimated before the coupling of artificial intelligence and the energy industry has been achieved:

(1) $$Y_{0it}=\delta_{t}+\theta_{t}Z_{i}+\lambda_{t}\mu_{i}+\varepsilon_{it}$$

where $\delta_{t}$ is the time fixed effect that affects the development of the energy industry in all provinces. The $Z_{i}$(*K* × 1) dimension covariable means that the observable control variable was not affected by the coupling effect between artificial intelligence and the energy industry. $\theta_{t}$ is a (1 × K)-dimensional unknown parameter vector. $\lambda_{t}$ is a common factor vector with (1 × F) dimensions that cannot be observed. $\mu_{i}$ is a (F × 1)-dimensional coefficient vector, representing the unobservable regional fixed effect. The error term $\varepsilon_{it}$ is a short-term shock that cannot be observed in every province. It was assumed that the mean value of $\varepsilon_{it}$ was 0 at the regional level. This model does not require independence between $Z_{i}$, $\mu_{i}$, and $\varepsilon_{it}$.

When $\lambda_{t}$ is a fixed constant—i.e., when the effect of unobservable influencing factors does not change over time—Equation (1) becomes a difference in difference (DID) model. The synthetic control method allows the influence of unobservable factors to change with time, i.e., $\lambda_{t}$ is a function of time *t*, rather than a constant term.

To calculate the effect of coupling artificial intelligence and the energy industry on the comprehensive development level of the energy industry, it is necessary to determine if the first province reached the comprehensive development level $Y_{01t}$ of the energy industry under the two-system coupling scenario. An (*N* × 1) dimensional weight vector $W=(w_{2},\cdot \cdot \cdot ,w_{N+1})$ was introduced, $w_{j}\geq 0$, j=2,···,N+1, which satisfied $w_{2}+\cdot \cdot \cdot +w_{N+1}=1$. When $w_{j}\geq 0$, a convex combination of reference group provinces was used to synthesize a “counterfactual reference group” to avoid a possible deviation. Each specific value of the vector *W*, which is an average weighting of all the regions within the reference group, represented a synthetic control over the first region. When the variable values of each reference group region were weighted, we obtained:

(2) $$\sum_{j=2}^{N+1}w_{j}Y_{jt}=\delta_{t}+\theta_{t}\sum_{j=2}^{N+1}w_{j}Z_{j}+\lambda_{t}\sum_{j=2}^{N+1}w_{j}\mu_{j}+\sum_{j=2}^{N+1}w_{j}\varepsilon_{jt}$$

Assuming that there is a weight vector $W^{*}=\left(w_{2}{}^{*},\cdot \cdot \cdot ,w_{N+1}{}^{*}\right)$, this satisfies:

(3) $$\sum_{j=2}^{N+1}w_{j}*Y_{j1}=Y_{11},\sum_{j=2}^{N+1}w_{j}*Y_{j2}=Y_{12},\cdot \cdot \cdot ,\sum_{j=2}^{N+1}w_{j}*Y_{jT0_{\;}}=Y_{1T0_{\;}},\sum_{j=2}^{N+1}w_{j}*Z_{j}=Z_{1}$$

Abadie et al. [46] proved that if $\sum_{t=1}^{T0_{\;}}\lambda_{t}^{\prime }\lambda_{t}$ is a nonsingular matrix, then:

(4) $$Y_{01t_{\;}}-\sum_{j=2}^{N+1}w_{j}*Y_{kt}=\sum_{j=2}^{N+1}w_{j}*\sum_{s=2}^{T_{0}}\lambda_{t}{\left(\sum_{n=1}^{T_{0}}\lambda_{n}^{\prime }\lambda_{n}\right)}^{-1}\lambda_{s}^{\prime }(\varepsilon_{js}-\varepsilon_{1s})-\sum_{j=2}^{N+1}w_{j}*(\varepsilon_{jt}-\varepsilon_{1t})$$

Abadie et al. [46] proved that under general conditions, Equation (4) approaches 0. Therefore, for $T_{0}<t\leq T$, the unbiased estimate of the counterfactual result for the first province could be approximated by the synthetic control group, i.e., ${\hat{Y}}_{01t}=\sum_{j=2}^{N+1}w_{j}*Y_{jt}$. The estimated value of the coupling effect on the comprehensive development level of the local energy industry was obtained when artificial intelligence and the energy industry were coupled:

(5) $${\hat{\tau }}_{1t}=Y_{1t}-\sum_{j=2}^{N+1}w_{j}*Y_{jt},t\in [T_{0+1},\cdot \cdot \cdot ,T]$$

The key to solving ${\hat{\tau }}_{1t}$ was to find the synthetic control vector $W^{*}=\left(w_{2}{}^{*},\cdot \cdot \cdot ,w_{N+1}{}^{*}\right)$ that makes Equation (3) true. The weight vector W^*^ was determined by minimizing the distance $\left|X_{1}-X_{0}W\right|$ between *X*_1_** and *X*_0_*W*. The distance function between *X*_1_** and *X*_0_*W* is expressed as ${\left\|X_{1}-X_{0}W\right\|}_{v}=\sqrt{{\left(X_{1}-X_{0}W\right)}^{\prime }V\left(X_{1}-X_{0}W\right)}$. *X*_1_** is the (m × 1)-dimensional feature vector of the first province before the coupling between artificial intelligence and the energy industry was realized. *X*_0_** is an (m × N)-dimensional matrix, and the *j*-th column of *X_0_* is the feature vector before the coupling of artificial intelligence and the energy industry in region *j*. *V* is a positive semidefinite matrix with (m × n) dimensional symmetry. To avoid the influence of *V* selection on the estimated mean square error of the model, the program developed by Abadie et al. [46] was used to calculate *V*, which resulted in the comprehensive development level of the energy industry in a synthetic area approximate to the comprehensive development level of the local energy industry when the coupling of the two systems was not realized. The comprehensive development level of the energy industry in the synthetic control area, which was obtained by weighting, could be used as an unbiased estimate of the comprehensive development level of the energy industry in a hypothetical area without the coupling of artificial intelligence and the energy industry. The difference in the comprehensive development level of the energy industry between the regions where artificial intelligence and the energy industry were coupled and the synthetic regions that were derived from the calculation reflected the coupling effect of the two systems on the comprehensive development level of the local energy industry.

### 3.2. Variable Selection

#### 3.2.1. Variable Descriptions and Data Sources

In this study, the comprehensive development index of the energy industry in 30 provinces of China from 2000 to 2017 was calculated using a comprehensive development index of the energy industry. This enabled changes in the comprehensive development of the regional energy industry to be measured. This value was then used as the prediction result variable in the synthetic control method. The comprehensive development index of the energy industry and artificial intelligence is shown in Table 1 and Table 2, and the comprehensive development index of the energy industry for the 30 provinces is shown in Table A1 of the Appendix A.

This paper mainly adopts the method of literature analysis to establish the evaluation index system of energy industry. The frequency analysis is carried out for the literature about the comprehensive index system of energy industry in China Knowledge Network, and the relevant evaluation indexes of energy industry used in recent years with high frequency and the structure division way of index system are selected. Combined with data availability, the following 10 indicators were finally selected to construct a comprehensive evaluation index system of China’ energy industry from three levels: total, structure and quality of the energy industry. Although the automation index can reflect the development of energy industry, it is difficult to obtain data, so it cannot be included in this index. China’ energy industry includes five sub-sectors: coal mining and processing, oil and natural gas extraction, petroleum processing and coking, electricity and steam, and hot water production and supply. Some provinces lack industrial investment data; therefore, the coal mining and washing industry and the oil and natural gas mining industry were combined into one indicator, referred to as the coal and oil mining industry. The unit industrial added value was calculated from the ratio of the terminal energy consumption to the industrial added value, and the power processing conversion rate was calculated from the input and output data of thermal power in the energy balance sheet.

No scientific research paper has yet measured the comprehensive development status of AI through the method of constructing an evaluation index system. With reference to the data analysis reports related to AI released by major consulting firms and considering the extremely low availability of AI-related data, the preliminary construction of a comprehensive evaluation index system for the development status of AI is revised. The comprehensive development status of AI in China is measured from three levels: public attention, technology and science education level, and market attention. Among them, the number of high-level papers refers to the geometric mean number of papers presented at top international academic conferences in AI, which eliminates the influence of the size of the number of paper co-authors on the average level of published high-level papers, and the number of pages must be greater than or equal to 6 pages to be counted. The number of high-level scholars is defined as scholars who have presented at least one high-level paper at a top academic conference in AI. The investment amount metric is derived from statistical analysis of data related to financing events obtained by AI companies. The investment amount of US dollar funds is converted using the average value of the exchange rate of US dollar to RMB in the current year. The known investment amounts of the investment rounds were weighted and averaged to obtain the weighted average of the amount invested in each round, which was used as the financing amount of undisclosed information. The final amount of investment received by AI companies was obtained. 

The economic indicators mentioned above were all converted by the GDP deflator to eliminate the impact of inflation. The standard coal reference coefficients of various energy sources involved in the calculation process were determined with reference to the standards given in the China Energy Statistics Yearbook. The conversion coefficients of coal, oil, and natural gas were 0.7143 kg of standard coal/kg, 1.45 kg of standard coal/kg, and 1.33 kg of standard coal/m^3^, respectively. The data related to the energy industry covered in this paper were obtained from the National Bureau of Statistics, the China Energy Statistical Yearbook from 2001–2018, and the regional statistical yearbooks of provinces and cities from 2001–2018. The data related to artificial intelligence are obtained from Baidu, INNOJOY patent search engine, CSRankings website, Hit Database, and Enterprise database of IT oranges.

To guarantee the fitting effect of the synthetic control object and the robustness of the result, several key factors that affected the development of the regional energy industry were included as predictive control variables, i.e., per capita GDP (*pgdp*), level of education per capita (*pedu*), fiscal spending as a share of GDP (*gov*), foreign investment as a share of GDP (*fi*), industrial structure (*ind*), and the level of science and technology (*tec*). The per capita GDP was used to measure the level of economic development of a region [54]. The level of education per capita was represented by the proportion of employed people with college level or higher education in the region [55], which reflected both the regional educational level and the quality of human resources. The ratio of fiscal expenditure to GDP was used to measure the government’s support for the market [56]. The ratio of foreign investment to GDP was used to reflect the impact of foreign technology on Chinese enterprises [57], and the industrial structure was expressed as the ratio of the added value of the regional secondary industry to GDP [58]. The level of science and technology was expressed by the number of regional patent applications granted [59]. To improve the fitting effect of the synthetic control method and the accuracy of the model, the lagged term of the comprehensive development index of the regional energy industry was considered part of the control variable by referring to Delaney and Kearney [60].

The entropy method was used to calculate the comprehensive development index of the energy industry. A comprehensive evaluation model of the energy industry was constructed based on the comprehensive evaluation index of the energy industry shown in Table 1. The detailed calculation formula was as follows:

(6) $$E(x)=\sum_{i=1}^{7}\sum_{j=1}^{10}w_{j}*X_{ij}^{\prime }\;\;i\;=\;1,\cdots ,7;j\;=\;1,\cdots ,10$$

where *E(x)* represents the comprehensive development index of the energy industry, *w_j_* is the weight of the *j*-th index calculated by the entropy weight method, and *X′_ij_* is the normalized value of a sample used in the entropy weight method.

The index data standardization method was as follows. Positive indexes refer to evaluation indexes where larger values are considered better. For positive indicators, the standardized treatment formula (7) was used:

(7) $$X_{ij}^{\prime }=\frac{X_{ij}-\min\left\{X_{1j},\cdots ,X_{nj}\right\}}{\max\left\{X_{1j},\cdots ,X_{nj}\right\}-\min\left\{X_{1j},\cdots ,X_{nj}\right\}}+0.001$$

Reverse indexes refer to evaluation indexes where smaller values are considered better. The standardized processing formula (8) was adopted for the reverse indexes:

(8) $$X_{ij}^{\prime }=\frac{\max\left\{X_{1j},\cdots ,X_{nj}\right\}-X_{ij}}{\max\left\{X_{1j},\cdots ,X_{nj}\right\}-\min\left\{X_{1j},\cdots ,X_{nj}\right\}}+0.001\;$$

Because the entropy method contains the calculation link of the logarithm in the solution process, a value of 0.001 was added to the data obtained after standardization to avoid a 0 value in the standardized data.

The evaluation index data related to the energy industry of all provinces in China from 2000 to 2017 were calculated from the China Energy Statistical Yearbook from 2001 to 2018 and the regional statistical yearbooks released by all provinces. The relevant data for the six predictive control variables were obtained from the National Bureau of Statistics, China Statistical Yearbook, China Population and Employment Statistical Yearbook, and China Labor Statistical Yearbook from 2001 to 2018.

#### 3.2.2. Selection of Cities in the Experimental Group

According to the comprehensive development index of the energy industry of 30 provinces in China from 2000 to 2017, among the five representative provinces where artificial intelligence is concentrated Guangdong has the highest level of comprehensive development in the energy industry, while Jiangsu was ranked second. The comprehensive development level of the energy industry in Beijing and Shanghai was the lowest level among the 30 provinces, which was consistent with their geographical location, resource endowment, and economic development plan. The comprehensive development level of the energy industry in Zhejiang was in the middle to lower level among the 30 provinces, with a small improvement over the 18 years. 

In summary, Guangdong and Jiangsu were selected as the experimental group provinces to test the effect of the coupled development of artificial intelligence and the energy industry on the local energy industry, and the results obtained were then considered convincing. Beijing, Shanghai, and Zhejiang, whose energy industries have been affected by the local development of artificial intelligence to varying degrees, were removed from the reference group of Guangdong and Jiangsu. The final reference group included 26 other regions, excluding Beijing, Shanghai, Zhejiang, and Xizang.

#### 3.2.3. Selection of Time Points

The coupled development of artificial intelligence and the energy industry had two levels: coupling and coupling coordination. To determine whether the two systems of artificial intelligence and the energy industry could be coupled to the development of the local energy industry after the coupling or the coupling and coordinated development were achieved, time points for both the coupling of the two systems and the coupling coordination of the two systems were selected.

The coupling degree, coordination degree, and stage distribution of artificial intelligence and the energy industry in China are shown in Table 3. A value of 0.5 was taken as the threshold value of both the coupling degree and coupling coordination degree. When the coupling degree of regional artificial intelligence and the energy industry exceeded 0.5, it was considered that the development of the two systems has reached a stable coupling stage. Similarly, when the coupling coordination degree of the two systems exceeded 0.5, it was considered that the two systems had reached a stable coupling and coordination stage. According to the coupling degree measurements presented in Table 1 of the Appendix A, the coupling degree of artificial intelligence and the energy industry in Guangdong exceeded the threshold value of 0.5 for the first time in 2012, and the coupling coordination degree of the two systems exceeded the threshold value of 0.5 in 2015. Therefore, the selected time points for the coupling effect in Guangdong were 2012 and 2015, respectively. The coupling effect on the energy industry could only be evaluated when the degree of integration between artificial intelligence and the energy industry was determined by comparing the synthetic results. 

From 2011 to 2017, the coupling degree of artificial intelligence and the energy industry in Jiangsu was higher than 0.5. However, according to the analysis, the coupling degree of the two systems in Jiangsu fluctuated, with an initial decrease and then an increase, with 2013 being a turning point. Therefore, 2013 was selected as the time point for a stable coupling between artificial intelligence and the energy industry in Jiangsu. Similarly, although the coupling coordination degree between artificial intelligence and the energy industry in Jiangsu exceeded the threshold value of 0.5 in 2011, eventually reaching 0.55, the coordination level only remained stable for one year, i.e., 2011. After four years, the coupling coordination degree improved steadily and exceeded 0.5 again in 2016. The trend of continuous improvement continued in 2017. In summary, 2016 was selected as the time point for the two systems to achieve stable coupling and coordinated development.

## 4. Analysis of the Empirical Results 

In this study, the synthetic control method was used to assess what kind of influence the development of artificial intelligence has on the comprehensive development level of the local energy industry. The study considered whether the scientific and technological changes resulting from the development of artificial intelligence after the coupling of artificial intelligence and the energy industry in specific regions will affect the comprehensive development level of the local energy industry and the degree of the coupling effect on the comprehensive development level of the regional energy industry.

### 4.1. Impact of the Coupling of Artificial Intelligence and the Energy Industry on the Integrated Development of the Energy Industry in Guangdong

Taking Guangdong as an example, this section uses the synthetic control method to explore the direction and intensity of the integrated development of artificial intelligence and energy industry on the comprehensive development level of local energy industry.

#### 4.1.1. Empirical Analysis of the Synthetic Control Method

Two different experimental results were obtained by the synthetic control method for 2012 and 2015 below. Table 4 presents the weight of each region in the synthetic Guangdong group. Table 5 shows the comparison between the fitting value and the true value of Guangdong predictive variables.

In the synthetic control method, the weight of provinces indicates the degree of contribution of each reference group province in the synthetic control group. The greater the weight, the greater the contribution of the reference group to the synthetic control object, and the more similar the characteristics of the synthetic group. A weight of 0 means that the similarity of the feature attributes between the reference group and the experimental group province is very low [47]. It can be seen from Table 4 that when the coupling effect occurred in 2012, the provinces with a relatively large weight in the synthetic Guangdong group were Ningxia, Henan, and Anhui. When 2015 was taken as the time point at which the coupling effect occurred, the provinces with a relatively large weight in the synthetic Guangdong group were Chongqing, Shandong, and Ningxia.

The root mean square percentage error (RMSPE) was used to measure the degree of fitting between a region and its synthetic control object. If the RMSPE value of a region was large before the coupling effect occurred, the fitting effect was not good [48]. It can be seen from Table 5 that when the coupling effect occurred in 2012, the RMSPE of the synthetic control method was 0.003077, and in 2015 it was 0.008373. In terms of RMSPE, 2012 was a better time point than 2015 for the coupling effects to take effect.

**Figure 1:**
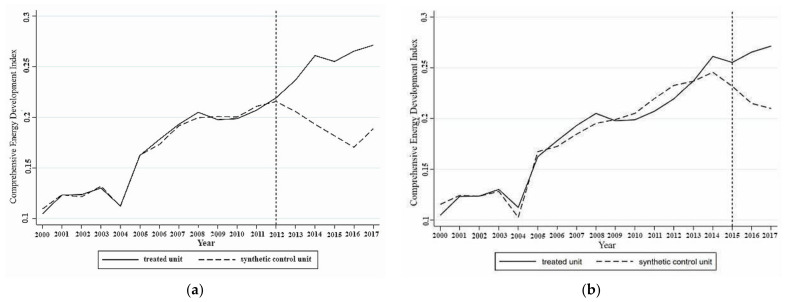
Comparison of actual and synthetic energy industry composite index growth in Guangdong in 2012 (**a**) and 2015 (**b**).

When 2012 was set as the time point when the coupling effect occurred, a comparison of the comprehensive development index of the energy industry of actual and synthetic Guangdong from 2000 to 2017 was conducted and is shown in Figure 1. Before 2012, the difference between the actual and synthetic values was 1.58%. The changes in the actual and synthetic values of the comprehensive development index of the energy industry in Guangdong almost completely coincided, indicating that the synthetic control method effectively replicated the growth path of the Guangdong energy industry comprehensive development index before the coupling effect occurred. When 2015 was used as the time point when the coupling effect occurred, the difference between the comprehensive development index of the energy industry in actual and synthetic Guangdong increased to 3.12%. With reference to Figure 1, the fitting effect of the synthetic control method was poor at this time. Before 2015, there was a certain deviation between the actual and synthetic Guangdong, and Figure 1 indicates that 2015 was not the exact time when the actual and synthetic values began to diverge.

In conclusion, the fitting effect was better when the coupling effect occurred in 2012. With these results more perfectly replicating the development path of the comprehensive development index of the energy industry in Guangdong before the coupling effect occurred. This result also shows that as long as artificial intelligence and the energy industry reach a stable coupling stage, the coupling effect can play a role in the development of the local energy industry. It is not necessary to realize the coordinated development of the two systems. Therefore, the further analysis focused on 2012 only.

#### 4.1.2. The Influence of the Coupling of Artificial Intelligence and the Energy Industry on the Energy Industry in Guangdong 

The effect of the coupling of artificial intelligence and the energy industry on the local energy industry, as estimated by the synthetic control method, was expressed by the degree of increase or decrease in Guangdong’s comprehensive development index of the energy industry relative to its synthetic control object after the coupling of the two systems was achieved. As shown in Figure 1a, when artificial intelligence and the energy industry in Guangdong reached a stable coupling in 2012, the development path of the actual and synthetic values of the comprehensive development index of the energy industry in Guangdong began to diverge. The actual value of the comprehensive development index of the energy industry in Guangdong became higher than the synthetic value, and the gap continued to widen. From 2013 to 2017, the comprehensive development index of the energy industry in Guangdong was 0.0005, 0.0268, 0.0634, 0.0686, 0.0903, and 0.0789 higher than that of synthetic Guangdong, representing increases of 0.24%, 12.74%, 32.04%, 36.76%, 51.57%, and 40.92%, respectively. From 2012 to 2017, compared with the synthetic value, the average annual growth rate of the actual value of the comprehensive development index of the energy industry in Guangdong was 29.05%. This result shows that after regional artificial intelligence and the energy industry reached a stable coupling stage, the coupling effect of the integration of the two systems substantially improved the comprehensive development level of the energy industry, with an average annual increase of 29.05%.

#### 4.1.3. Validity Test

Although the empirical results of the synthesis control method showed that there were significant differences between the actual and synthetic values of the comprehensive development index of Guangdong’s energy industry, it was not clear whether this difference was caused by the coupling effect of the two systems or whether the difference was accidental. The following proof was used to test the validity of the result. 

##### Placebo Test

With reference to the placebo test method in the robustness test proposed by Abadie and Gardeazabai [45] and Abadie et al. [46], the following methodology was adopted. Regions in the sample period that had not realized the coupling of artificial intelligence and the energy industry were selected for analysis alongside Guangdong. If the comprehensive development index of the energy industry in an area was the same as the development trend of Guangdong, the results obtained by the synthetic control method for Guangdong were not considered reliable. Therefore, it could not be proven that the coupling of artificial intelligence and the energy industry promoted the development of the regional energy industry. In contrast, if the comprehensive development index of the energy industry in this region after 2012 differed from, or was even the opposite of, the development trend of Guangdong, the results were considered to be robust. A placebo test was conducted in the provinces with the largest weight and provinces with a weight of 0 in the synthetic Guangdong group. If these two extreme cases showed different development trends from that of Guangdong, the robustness of the results were proven.

**Figure 2:**
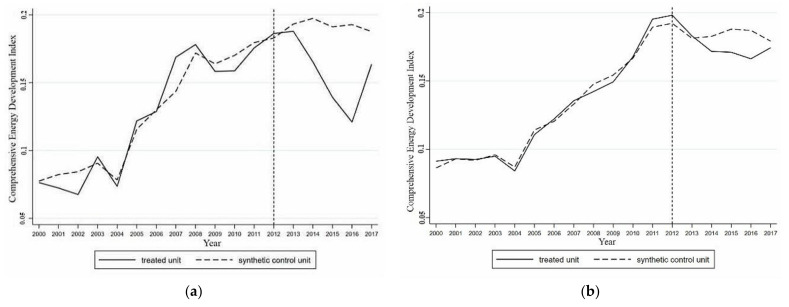
Placebo test in Guangdong. (**a**) The comprehensive development index of energy industry in Ningxia and synthetic Ningxia; (**b**) The comprehensive development index of energy industry in Hubei and synthetic Hubei.

A placebo test was conducted for Ningxia with the highest weight and Hubei with a weight of 0 in the synthetic Guangdong group, and the results are shown in Figure 2. It can be seen that after 2012, the actual value of the comprehensive development index of the energy industry in Ningxia displayed a contrasting trend to that of Guangdong. The actual value of the comprehensive development index of the energy industry in Hubei was similar to the trend observed for the synthetic control object. According to the above placebo test, when the two systems were coupled in 2012, the coupled artificial intelligence and energy industry in Guangdong played a significant role in promoting the development of the local energy industry, and this result was robust.

##### Ranking Test

To prove that the results of Guangdong’s synthetic control method were statistically significant, the ranking test method proposed by Abadie et al. [46] was applied. The ranking test was used to determine the probability of the same situation as that in Guangdong occurring in other provinces. The basic concept was as follows. It was assumed that the artificial intelligence and energy industries of all provinces in the reference group realized the coupled development of the two systems in 2012. The synthetic control method was then used to construct the synthetic control objects of the reference group provinces. By comparing the degree of difference between the coupling effect of the two systems when coupled development was realized and the actual coupling effect of Guangdong, if the coupling effect of the two systems in Guangdong was large enough, it was reasonable to accept that the results of the synthetic control in Guangdong were not accidental and were statistically significant. The coupling of artificial intelligence and the energy industry in Guangdong had a coupling effect on the development of the local energy industry.

**Figure 3:**
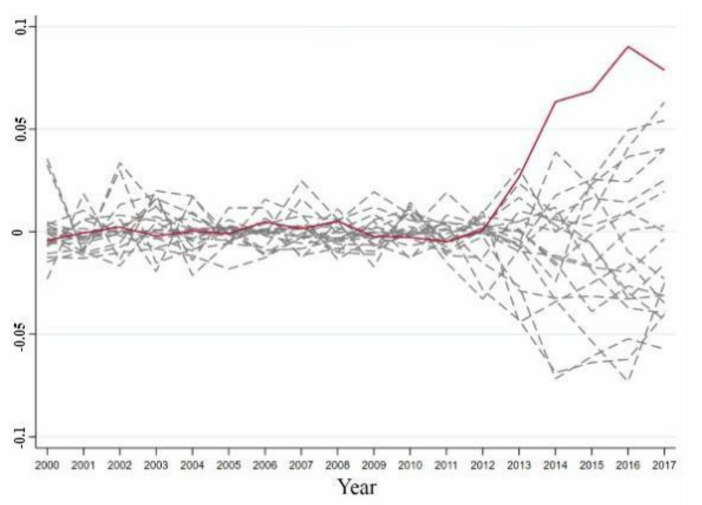
Difference distribution of the comprehensive development index of energy industry of the provinces in the Guangdong’s reference group. Note: The red solid line represents Guangdong, and the gray dotted line represents the provinces where the RMSPE value is 5 times lower than the Guangdong RMSPE value.

According to the treatment method of Abadie et al. [46], provinces with a poor fitting effect before 2012 should be excluded to prove the effectiveness of the synthetic control method. Provinces with an RMSPE more than five times that of Guangdong before 2012 were removed, leaving a total of 19 provinces. The reason for this was that before 2012, the synthetic control objects did not fit the trend of the comprehensive development index of the energy industry in the region, and the difference between the actual value of the comprehensive development index of the energy industry in the region and the predicted value after 2012 was probably caused by a bad fitting effect. It was not likely that this was related to the coupling of artificial intelligence and the energy industry. Figure 3 shows the distribution of the differences between the actual and synthetic values of the reference provinces, which were five times lower than the RMSE of Guangdong.

Figure 3 shows that before 2012, the difference between the actual and synthetic values of the comprehensive development index of the energy industry in Guangdong was smaller than the differences based on the comprehensive development index values of the energy industry in other provinces. However, since 2012, the difference between the actual and synthetic values in Guangdong has increased significantly, and the gap between the difference in Guangdong and the difference in other provinces has gradually widened. The gap between the difference in Guangdong was larger than the range of differences in other provinces. The difference between the actual and synthetic values in Guangdong was much larger than the gap between the actual and synthetic values in other provinces. The comprehensive development level of the energy industry in Guangdong has therefore significantly improved due to the impact of the coupled development of local artificial intelligence and the energy industry. There was only a 5.26% probability that there would be such a large difference between the comprehensive development index of the energy industry of actual and synthetic Guangdong. Due to the coupling of artificial intelligence and the energy industry in Guangdong, the substantial increase in the comprehensive development index of the energy industry in Guangdong was significant at the 10% level and the synthetic control method produced effective results. 

### 4.2. The Impact of the Coupling of Artificial Intelligence and the Energy Industry on the Energy Industry in Jiangsu 

Taking Jiangsu as an example, this section uses the synthetic control method to explore the direction and intensity of the integrated development of artificial intelligence and energy industry on the comprehensive development level of local energy industry.

#### 4.2.1. Empirical Analysis of the Synthetic Control Method

The results obtained by two different synthetic control methods in 2013 and 2016 are presented below.

According to the weight of each region in synthetic Jiangsu in Table 6, when 2013 was taken as the time point for the coupling effect to occur, the provinces with a relatively large weight in the synthetic Jiangsu group were Hunan, Qinghai, and Anhui. When 2016 was taken as the time point for the coupling effect to occur, the provinces with a relatively large weight in the synthetic Jiangsu group were Qinghai, Chongqing, and Shaanxi.

According to the comparison between the fitting value and the actual value of the predictive variables in Jiangsu in Table 7, when 2013 was taken as the time point for when the coupling effect in the synthetic control method occurred, the RMSPE value was 0.0026114. When 2016 was taken as the time point when the coupling effect in the synthetic control method occurred, the RMSPE value was 0.0065547. In terms of the RMSPE, the fitting effect was better when 2013 was used as the time point of the coupling effect in the synthetic control method.

**Figure 4:**
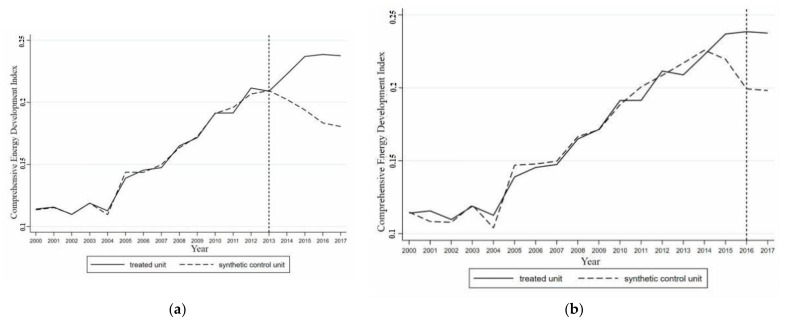
Comparison of actual and synthetic energy industry synthetic index growth in Jiangsu in 2013 (**a**) and 2016 (**b**).

Figure 4 shows a comparison between the growth of the synthetic and actual values of the comprehensive development index for Jiangsu’s energy industry from 2000 to 2017, taking 2013 as the time point when the coupling effect occurred. The results obtained by the synthetic control method better fitted the comprehensive development index of the energy industry in Jiangsu before the coupling effect occurred. The difference between the actual and synthetic values was only 1.25%, and the trend of the comprehensive development index of the energy industry in synthetic Jiangsu and actual Jiangsu were almost completely the same. The synthetic control method perfectly replicated the growth path of the comprehensive development index of the energy industry in Jiangsu before the coupling of the two systems. Taking 2016 as the time point when the coupling effect occurred, before 2016 the difference in the comprehensive development index of the energy industry between actual and synthetic Jiangsu was 2.96%, which was more than twice the difference in 2013. There was a deviation in the trend of the comprehensive development index of the energy industry between the actual and synthetic Jiangsu before 2016. The actual and synthetic values began to separate significantly before 2016. It can be seen that the time point of 2016 as the occurrence of the coupling of artificial intelligence and the energy industry was not accurate, with 2013 being more realistic. This was consistent with the conclusion obtained from the synthetic control method of Guangdong, i.e., once artificial intelligence and the energy industry reached a stable coupling stage, the coupling effect of the two systems on the development of the regional energy industry was exerted. Therefore, further analysis focused only on 2012.

#### 4.2.2. The Impact of the Coupling of Artificial Intelligence and the Energy Industry on the Energy Industry in Jiangsu

As shown in Figure 4, when artificial intelligence and the energy industry in Jiangsu reached a stable coupled stage in 2013, the actual value of the comprehensive development index of the energy industry in Jiangsu began to separate from the synthetic value. The actual value of the comprehensive development index of energy industry in Jiangsu became higher than the synthetic value, and the gap between the actual and synthetic values increased gradually. From 2014 to 2017, the actual value of comprehensive development index of the energy industry in Jiangsu was higher than the synthetic value. After the two systems achieved a stable coupling, the comprehensive development level of the energy industry in Jiangsu increased by 9.11% (2014), 20.24% (2015), 27.70% (2016), and 27.16% (2017). Since 2013, the comprehensive development level of Jiangsu’s energy industry has increased by 21.05% annually. Therefore, achieving a stable and coupled development between regional artificial intelligence and the energy industry could boost the comprehensive development level of the regional energy industry by 21.05% every year.

#### 4.2.3. Validity Test

To ensure the robustness and validity of the empirical results of the synthetic control method, the same robustness and validity tests were conducted in Jiangsu and Guangdong.

##### Placebo Test

**Figure 5:**
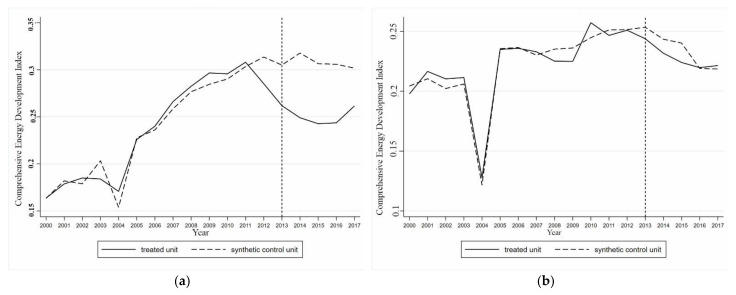
Placebo test in Jiangsu; (**a**) The comprehensive development index of energy industry in Hunan and synthetic Hunan. (**b**) The comprehensive development index of energy industry in Liaoning and synthetic Liaoning.

Placebo tests were conducted for Hunan, which had the highest weight in the synthetic Jiangsu group, and Liaoning, which had a weight of 0. The results are shown in Figure 5. It can be seen that the changes in the comprehensive development index of the energy industry in Hunan and Liaoning did not follow the same trend as in Jiangsu after 2013. In Hunan, there was a divergence between the actual and synthetic values of the comprehensive development index of the energy industry before 2013, with the actual value being lower than the synthetic value. This trend differed from the trend in Jiangsu. The actual and synthetic values of the comprehensive development index of the energy industry in Liaoning changed in a similar way, with no significant separation phenomenon. The placebo test results showed that artificial intelligence and the energy industry in Jiangsu achieved a stable coupling in 2013 and had a coupling effect on the local energy industry. This was an important reason for the significant increase in the comprehensive development level of the energy industry in Jiangsu after 2013.

##### Ranking Test

**Figure 6:**
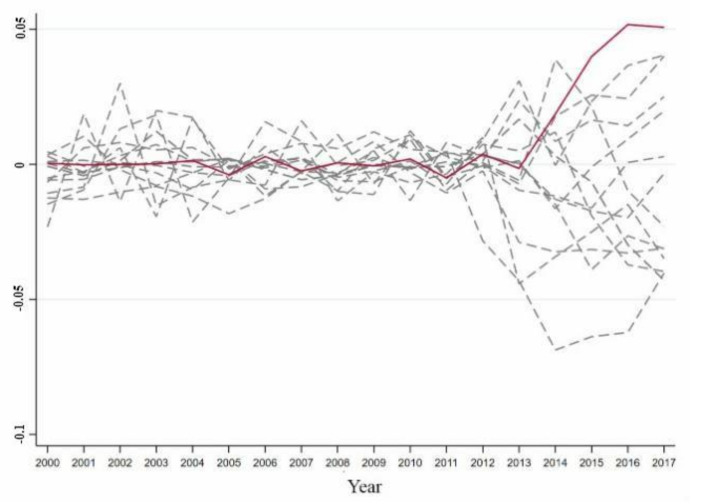
Difference distribution of the comprehensive development index of energy industry of the provinces in Jiangsu’s reference group; Note: The red solid line represents Jiangsu, and the gray dotted line represents the provinces where the RMSPE value is 5 times lower than the Jiangsu RMSPE value.

The provinces whose RMSPE exceeded that of Jiangsu by five times before 2013 were excluded from the analysis, leaving 14 provinces. Figure 6 shows the difference in the distribution between the actual and synthetic values for provinces in the reference group that were less than five times the RMSPE of Jiangsu. It can be seen that before 2013, including Jiangsu, the difference between the actual and synthetic values of the comprehensive development index of the energy industry in all the provinces that met the requirements was very small. Since 2013, the difference between the actual and synthetic values in Jiangsu has increased significantly, and differences have begun to appear in other provinces. The differences in Jiangsu were of a different magnitude to those in other provinces. This indicates that the difference between the actual and synthetic values in Jiangsu was much larger than that between the actual and synthetic values in other provinces. Therefore, because the local artificial intelligence and energy industry achieved a stable coupling, the comprehensive development level of the energy industry in Jiangsu has significantly improved. There was only a 7.14% probability that there would be such a large gap between the actual and synthetic values of the comprehensive development index of the energy industry of Jiangsu. The results showed that because a coupling of artificial intelligence and the energy industry occurred, the improvement of the comprehensive development index of energy industry in Jiangsu was significant at the 10% level, and the results obtained using the synthetic control method were valid.

## 5. Conclusions and Policy Implications

### 5.1. Conclusions

In order to scientifically and rigorously measure the comprehensive development level of AI and energy industry in China and the regions, and to explore the degree of influence of the integrated development of AI and energy industry on the comprehensive development level of regional energy industry, this paper firstly calculated the comprehensive development index of energy industry in 30 provinces and cities across China during 2000–2017 using the comprehensive evaluation index system of energy industry, and took Guangdong and Jiangsu as examples. The synthetic control method was used to explore the direction and impact intensity of the integration development of two systems of artificial intelligence and energy industry on the comprehensive development level of local energy industry, and the robustness and validity of the empirical results were verified by placebo and ranking tests. We eventually drew the following conclusions:

(1) When a stable coupling development relationship between regional AI and energy industry was achieved without the need to reach a coordination relationship, the coupling effect began to contribute to the improvement of the comprehensive development level of the local energy industry.

(2) After the stable coupling development between regional AI and energy industry, the comprehensive development level of energy industry in Guangdong and Jiangsu grew at more than 20% per year, and this empirical result was significant at 10% level.

(3) The placebo test results showed that the coupling development of the two systems between AI and energy industry in Guangdong Province was achieved in 2012, which promoted the development of local energy industry; The stable coupling development of the two systems between AI and energy industry in Jiangsu Province was achieved in 2013 and had a coupling effect on local energy industry, which was an important reason for the significant increase of the comprehensive development level of energy industry in Jiangsu Province after 2013.

### 5.2. Policy Implications

The synthetic control method predicted the values of the regional energy industry comprehensive development index when not affected by the coupling effect of the two systems, and quantified the magnitude of the coupling effect of the integral development of AI and energy industry in Guangdong and Jiangsu provinces on the comprehensive development level of the local energy industry by comparing the differences between the synthetic and actual values, which more concretely illustrated the far-reaching impact of the development of AI on the development of the energy industry and provided data support and theoretical basis for the regional practice of empowering traditional industries through AI.

Based on the model constructed and empirical findings of this paper, the following policy measures need to be improved to maximize the technological dividends of artificial intelligence and help China’s smart energy construction goals:

(1) Promote the development of the artificial intelligence industry in each region according to local conditions. According to the comprehensive development index accounted for in this paper, it can be seen that, currently, China’s artificial intelligence is generally at the stage of technology first, commercial implementation lags behind, and the development of serious imbalance between regions, artificial intelligence enterprises are more concentrated in many Internet enterprises and economically developed regions. The development of artificial intelligence has far-reaching significance in accelerating the structural change of traditional industrial chain, improving the depth of information mining, information utilization and production operation efficiency. To use artificial intelligence as an important engine of economic growth, local governments at all levels should actively respond to the national strategic goal of encouraging, supporting, and guiding the development of local artificial intelligence technology and enterprises. Combining the advantages of the local economy, science and technology, regional conditions, and environmental resources, the government should introduce preferential tax policies for artificial intelligence enterprises, policies to attract artificial intelligence professionals, and policies to develop the required skills. 

(2) The government, enterprises, and universities should cooperate to promote the coordinated development of artificial intelligence and the energy industry. The government and enterprises should work together to guide, establish, and improve the data ecosystem, and accelerate the innovation and upgrading of AI-related technologies and their application in the field of energy industry. Local enterprises should understand the development plans and strategic goals of national and local governments, grasp the development trend of artificial intelligence and energy industry, combine their own advantages, and find their deep field and future development direction. By conducting industry–university–research cooperation activities with universities and communicating with successful local and international artificial intelligence companies, enterprises can acquire the latest development trends, cutting-edge theories of artificial intelligence, and implement technology and business models to improve their own strengths.

(3) Formulate measures for the differentiated integration of artificial intelligence and the energy industry between regions. Due to the heterogeneity of the economic development level, geographical location, and resource endowment in different regions of China, there are significant differences in the integration of artificial intelligence and the energy industry in different provinces. In order to accelerate the integration of artificial intelligence with the regional energy industry, local governments at all levels and regional enterprises should identify their own advantages, determine a suitable positioning, and formulate goals and directions in line with their own development conditions and artificial intelligence development. Combining the key strengths and challenges of the local energy industry will increase innovation in the development of artificial intelligence-related technologies, product research and development, and the introduction of technology and skills. This will promote the integration of artificial intelligence and the energy industry, ease the consumption of energy resources, and solve the development dilemma in the energy industry.

## Figures and Tables

**Table 1:** Energy comprehensive evaluation index system.

First Level Indicator	Second Level Indicator	Third Level Indicator	Unit	Type
Energy comprehensive evaluation index system	Total amount index	Energy industry investment	Billion	Positive index
		Total energy production	10,000 tons standard coal	Positive index
		Total energy consumption	10,000 tons standard coal	Negative index
	Structural index	Proportion of investment in power, steam, and hot water production and supply industries	%	Positive index
		Proportion of investment in petroleum processing and coking industry	%	Positive index
		Proportion of investment in coal mining and selection, oil and gas extraction	%	Positive index
	Quality index	Coefficient of elasticity of energy consumption	--	Negative index
		Energy consumption per unit of GDP	10,000 tons of standard coal/100 million yuan	Negative index
		Energy consumption per unit of industrial value added	10,000 tons of standard coal/100 million yuan	Negative index
		Conversion rate of power energy processing	%	Positive index

**Table 2:** Artificial intelligence comprehensive evaluation index system.

First Level Indicator	Second Level Indicator	Third Level Indicator	Unit	Type
Artificial intelligence comprehensive evaluation index system	Public attention	Baidu Index	/	Positive index
	Science and education level	Number of patent applications	Number	Positive index
		Number of research projects	Number	Positive index
		Number of high-level papers	Number	Positive index
		Number of high-level scholars	people	Positive index
	Market attention	investment amount	100 million yuan	Positive index

**Table 3:** The coupling degree, coordination degree, and stage distribution of artificial intelligence and energy industry in China.

Year	E	I	Degree of Coupling	Comprehensive Evaluation Index	Coupling Coordination	Coupling Level	Coupling Coordination Level	Comprehensive Evaluation
2011	0.28	0.04	0.64	0.16	0.32	Moderate running-in stage	Initial coordination stage	Initial coordination and moderate running-in stage
2012	0.43	0.18	0.91	0.30	0.53	High coupling stage	Low coordination stage	Low coordination coupling stage
2013	0.54	0.12	0.78	0.33	0.51	Deep running-in stage	Low coordination stage	Low coordination depth running-in stage
2014	0.62	0.18	0.83	0.40	0.58	High coupling stage	Low coordination stage	Low coordination coupling stage
2015	0.61	0.37	0.97	0.49	0.69	High coupling stage	Mid-coordination stage	Coordination and coupling phase
2016	0.59	0.48	1.00	0.54	0.73	High coupling stage	Deep coordination stage	Deep coordination and coupling stage
2017	0.65	1.00	0.98	0.83	0.90	High coupling stage	Extremely coordinated stage	Extremely coordinated coupling stage

Notes: E represents energy industry composite index. I represents artificial intelligence composite index.

**Table 4:** The weight of each region in synthetic Guangdong.

Province	Ningxia	Henan	Anhui	Fujian	Hainan	Jilin	Xinjiang	Shanxi	-
2012	0.353	0.235	0.157	0.104	0.069	0.067	0.009	0.007	-
Province	Chongqing	Shandong	Ningxia	Fujian	Hainan	Neimenggu	Gansu	Xinjiang	Anhui
2015	0.222	0.181	0.154	0.128	0.121	0.069	0.067	0.05	0.008

**Table 5:** The comparison between the fitting value and the actual value of Guangdong predictive variables.

	2012		2015	
	Guangdong	Synthetic Guangdong	Guangdong	Synthetic Guangdong
RMSPE	0.003077		0.008373	
*lnpgdp*	10.1485	9.4597	10.3148	9.8198
*lnedu*	2.0182	1.8939	2.1402	2.0591
*lnind*	3.8868	3.8139	3.8798	3.7991
*lntec*	10.6760	7.3484	10.9467	7.9889
*lnfi*	−0.0163	−1.2304	−0.1446	−1.1845
*lngov*	2.3653	2.8503	2.4098	2.8266
*eicdi* in 2000	0.1047	0.1095	0.1047	0.1154
*eicdi* in 2004	0.1124	0.1120	0.1124	0.1026
*eicdi* in 2008	0.205	0.1997	0.205	0.1952
*eicdi* in 2010	0.1987	0.2004	0.1987	0.2050
*eicdi* in 2011	0.2071	0.2109	0.2071	0.2196
*eicdi* in 2012	--	--	0.2193	0.2325
*eicdi* in 2014	--	--	0.2611	0.2456

Note: *eicdi* represents the comprehensive development index of energy industry. The table below is the same.

**Table 6:** The weight of each region in synthetic Jiangsu.

Province	Hunan	Qinghai	Anhui	Shanxi	Fujian	Gansu	Hainan	Guangxi	Hubei
2013	0.317	0.273	0.101	0.098	0.056	0.046	0.046	0.038	0.025
Province	Qinghai	Chongqing	Shaanxi	Sichuan	Hainan	Shandong	Fujian		
2016	0.325	0.325	0.177	0.103	0.064	0.003	0.002		

**Table 7:** The comparison between the fitting value and the true value of Jiangsu predictive variables.

	2013		2016	
	Jiangsu	Synthetic Jiangsu	Jiangsu	Synthetic Jiangsu
RMSPE	0.0026114		0.0065547	
*lnpgdp*	10.2462	9.4492	10.4455	9.6837
*lnedu*	1.9997	1.9134	2.1791	2.0526
*lnind*	3.9837	3.7848	3.9596	3.8380
*lntec*	10.2569	7.3522	10.6477	7.5161
*lnfi*	−0.0607	−1.5502	−0.1186	−1.4735
*lngov*	2.2661	2.9852	2.3263	3.1700
*eicdi* in 2000	0.1141	0.1133	0.1141	0.1148
*eicdi* in 2004	0.1126	0.1095	0.1126	0.1039
*eicdi* in 2008	0.1649	0.1633	0.1649	0.1665
*eicdi* in 2010	0.1913	0.1911	0.1913	0.1884
*eicdi* in 2012	0.2115	*0.2067*	0.2115	0.2085
*eicdi* in 2014	--	--	0.2227	0.2258
*eicdi* in 2015	--	--	0.2369	0.2196

## Data Availability

Data available in a publicly accessible repository. The data presented in this study are openly available in the China Energy Statistical Yearbook from 2001 to 2018, the regional statistical yearbooks released by all provinces, the National Bureau of Statistics, China Statistical Yearbook, China Population and Employment Statistical Yearbook, and China Labor Statistical Yearbook from 2001 to 2018.

## Appendix A

**Table A1:** Comprehensive development index of the energy industry in 30 provinces across the country from 2000 to 2017.

	2000	2001	2002	2003	2004	2005	2006	2007	2008	2009	2010	2011	2012	2013	2014	2015	2016	2017
Shanxi	0.20	0.23	0.28	0.32	0.27	0.38	0.39	0.41	0.44	0.44	0.52	0.59	0.61	0.57	0.56	0.58	0.50	0.46
Shaanxi	0.12	0.15	0.16	0.20	0.19	0.23	0.24	0.25	0.29	0.33	0.36	0.41	0.43	0.46	0.44	0.44	0.41	0.43
Shandong	0.22	0.24	0.24	0.27	0.18	0.29	0.28	0.32	0.33	0.33	0.34	0.36	0.37	0.36	0.38	0.38	0.39	0.41
Hebei	0.13	0.13	0.15	0.15	0.12	0.20	0.20	0.22	0.23	0.23	0.25	0.26	0.28	0.28	0.27	0.27	0.28	0.29
Neimenggu	0.09	0.10	0.10	0.14	0.14	0.21	0.27	0.32	0.38	0.40	0.44	0.48	0.50	0.51	0.53	0.45	0.44	0.29
Xinjiang	0.23	0.31	0.22	0.24	0.09	0.27	0.28	0.29	0.28	0.28	0.29	0.29	0.31	0.33	0.37	0.35	0.29	0.29
Guangdong	0.10	0.12	0.12	0.13	0.11	0.16	0.18	0.19	0.20	0.20	0.20	0.21	0.22	0.24	0.26	0.26	0.27	0.27
Henan	0.16	0.18	0.19	0.18	0.17	0.23	0.24	0.27	0.28	0.30	0.30	0.31	0.29	0.26	0.25	0.24	0.24	0.26
Sichuan	0.10	0.10	0.11	0.12	0.11	0.14	0.16	0.17	0.18	0.20	0.22	0.26	0.26	0.24	0.25	0.26	0.25	0.26
Heilongjiang	0.25	0.24	0.25	0.25	0.12	0.26	0.27	0.27	0.28	0.28	0.29	0.30	0.29	0.28	0.26	0.26	0.24	0.25
Jiangsu	0.11	0.12	0.11	0.12	0.11	0.14	0.15	0.15	0.16	0.17	0.19	0.19	0.21	0.21	0.22	0.24	0.24	0.24
Guizhou	0.08	0.08	0.08	0.10	0.10	0.13	0.14	0.14	0.17	0.19	0.21	0.26	0.25	0.24	0.22	0.24	0.26	0.23
Liaoning	0.20	0.22	0.21	0.21	0.13	0.23	0.24	0.23	0.23	0.22	0.26	0.25	0.25	0.24	0.23	0.22	0.22	0.22
Yunnan	0.07	0.07	0.08	0.09	0.07	0.12	0.12	0.13	0.14	0.15	0.16	0.18	0.20	0.22	0.21	0.22	0.21	0.20
Tianjing	0.18	0.20	0.19	0.21	0.08	0.22	0.22	0.20	0.23	0.24	0.24	0.21	0.19	0.22	0.23	0.21	0.17	0.18
Anhui	0.11	0.16	0.13	0.17	0.15	0.18	0.19	0.19	0.20	0.22	0.21	0.22	0.23	0.20	0.19	0.19	0.18	0.18
Hunan	0.09	0.10	0.09	0.09	0.09	0.13	0.12	0.14	0.16	0.18	0.21	0.22	0.23	0.23	0.22	0.20	0.19	0.18
Zhejiang	0.08	0.09	0.10	0.10	0.08	0.11	0.11	0.12	0.12	0.12	0.13	0.13	0.14	0.15	0.16	0.17	0.18	0.18
Hubei	0.09	0.09	0.09	0.09	0.08	0.11	0.12	0.14	0.14	0.15	0.17	0.20	0.20	0.18	0.17	0.17	0.17	0.17
Jilin	0.12	0.15	0.16	0.17	0.06	0.17	0.20	0.19	0.18	0.20	0.20	0.20	0.22	0.19	0.20	0.21	0.19	0.17
Fujian	0.07	0.08	0.12	0.08	0.06	0.09	0.11	0.15	0.16	0.15	0.17	0.16	0.17	0.18	0.17	0.17	0.17	0.17
Ningxia	0.08	0.07	0.07	0.10	0.07	0.12	0.13	0.17	0.18	0.16	0.16	0.18	0.19	0.19	0.17	0.14	0.12	0.16
Chongqing	0.07	0.07	0.08	0.08	0.05	0.11	0.11	0.12	0.14	0.15	0.15	0.17	0.17	0.18	0.21	0.20	0.16	0.15
Gansu	0.10	0.10	0.11	0.12	0.11	0.14	0.14	0.13	0.13	0.15	0.15	0.16	0.18	0.20	0.19	0.19	0.16	0.15
Guangxi	0.06	0.06	0.06	0.07	0.05	0.08	0.09	0.10	0.13	0.12	0.13	0.14	0.13	0.14	0.13	0.14	0.13	0.14
Jiangxi	0.07	0.07	0.08	0.09	0.06	0.10	0.09	0.10	0.13	0.13	0.12	0.15	0.15	0.15	0.14	0.12	0.13	0.12
Qinghai	0.16	0.12	0.12	0.13	0.10	0.14	0.13	0.13	0.14	0.12	0.15	0.12	0.13	0.14	0.14	0.13	0.12	0.12
Shanghai	0.09	0.12	0.11	0.09	0.09	0.08	0.09	0.12	0.13	0.11	0.11	0.12	0.13	0.09	0.09	0.09	0.09	0.10
Beijing	0.08	0.07	0.06	0.06	0.07	0.09	0.09	0.09	0.11	0.10	0.09	0.09	0.09	0.09	0.09	0.08	0.08	0.09
Hainan	0.11	0.11	0.09	0.06	0.13	0.18	0.17	0.07	0.06	0.08	0.06	0.08	0.10	0.11	0.11	0.10	0.10	0.09
